# MiR-30a and miR-200c differentiate cholangiocarcinomas from gastrointestinal cancer liver metastases

**DOI:** 10.1371/journal.pone.0250083

**Published:** 2021-04-14

**Authors:** Jun Won Park, Jong Min Jeong, Kye Soo Cho, Soo Young Cho, Jae Hee Cheon, Dong Ho Choi, Sang Jae Park, Hark Kyun Kim

**Affiliations:** 1 National Cancer Center of Korea, Goyang, Republic of Korea; 2 Department of Biomedical Convergence, Kangwon National University, Kangwon, Republic of Korea; 3 Department of Internal Medicine and Institute of Gastroenterology, Yonsei University College of Medicine, Seoul, Republic of Korea; 4 Departments of Surgery, Hanyang University School of Medicine, Seoul, Republic of Korea; University of Oklahoma Health Sciences Center, UNITED STATES

## Abstract

Prior studies have demonstrated the utility of microRNA assays for predicting some cancer tissue origins, but these assays need to be further optimized for predicting the tissue origins of adenocarcinomas of the liver. We performed microRNA profiling on 195 frozen primary tumor samples using 14 types of tumors that were either adenocarcinomas or differentiated from adenocarcinomas. The 1-nearest neighbor method predicted tissue-of-origin in 33 samples of a test set, with an accuracy of 93.9% at feature selection p values ranging from 10^−4^ to 10^−10^. According to binary decision tree analyses, the overexpression of miR-30a and the underexpression of miR-200 family members (miR-200c and miR-141) differentiated intrahepatic cholangiocarcinomas from extrahepatic adenocarcinomas. When binary decision tree analyses were performed using the test set, the prediction accuracy was 84.8%. The overexpression of miR-30a and the reduced expressions of miR-200c, miR-141, and miR-425 could distinguish intrahepatic cholangiocarcinomas from liver metastases from the gastrointestinal tract.

## Introduction

Being able to predict where a metastatic tissue originated from is important for the clinical management of patients with metastatic cancers, and microRNA profiling has been used successfully to predict the tissue-of-origin for metastases [1–5]. Rosenfeld et al. reported a prediction accuracy of 89% using the first-generation of Rosetta Genomics microRNA assays [2]. Using the second-generation of these assays, Meiri et al. reported an 85% overall accuracy, and a 90% sensitivity for single-answer cases in an independent sample set [3]. Using assays based on 47 microRNAs, Ferracin et al. reported prediction accuracies of 100% and 78% for primary cancers and metastases, respectively [4]. Sokilde et al. reported that their 132 microRNA-based assays correctly predicted tissue-of-origin in 88% of metastases [5]. While these prior microarray studies have demonstrated the utility of microRNA assays for predicting cancer tissue-of-origin, these assays need to be further optimized to predict tissue-of-origin for liver metastases.

Being able to identify liver as the source, and to identify liver cancer by type, is important because the liver is a common site for cancer metastasis. It is therefore clinically important to distinguish cancer metastases from primary liver cancer to plan for optimal patient care. Whereas hepatocellular carcinoma (HCC) is easily distinguished from liver metastases by histology, intrahepatic cholangiocarcinoma, which represents 4–6% of primary liver cancers, is often difficult to differentiate from metastatic adenocarcinoma [6]. Histologically, primary intrahepatic cholangiocarcinomas are adenocarcinomas that resemble the metastases of common solid tumors such as colorectal or pancreatic adenocarcinomas [7, 8]. Being able to distinguish between these two disease entities is important because the treatment plans and prognoses are different, and there are no specific immunohistochemistry markers for intrahepatic cholangiocarcinomas. According to Chiu et al., CK7-positive/CK20-negative staining was seen in 9 out of 12 (75%) cholangiocarcinomas, but in none of the 25 colorectal cancer metastases examined, whereas CK7-negative/CK20-positive staining was seen in 1 out of 12 (8%) cholangiocarcinomas, and in 20 out of 25 (80%) colorectal cancer metastases [8]. The sensitivity of CDX2 for colorectal cancer is 99%, but CDX2 is also expressed in up to 21% of intrahepatic cholangiocarcinomas [9, 10]. Even microRNA-based assays have only demonstrated relatively weak performance for predicting tissue-of-origin for digestive system cancers, especially for cholangiocarcinomas. The microRNA classifiers of Solkide et al. failed to predict tissue-of-origin for most liver metastases, but did classify them as cholangiocarcinomas [5]. Therefore, these authors had to add a rule to their classifier model that metastasis sites cannot be classified as primary tumors [5]. Although cholangiocarcinomas have been included to the second-generation Rosetta Genomics microRNA assay, 4 of 13 biliary tract cancers (30.8%) were either misclassified, or ambiguously predicted to be pancreatobiliary cancers using it [3]. In addition, 5 of 9 pancreatic adenocarcinomas (55.5%) were either misclassified, or ambiguously predicted to be pancreatobiliary cancers [3]. There has been one microRNA study focused on comparing pancreatic cancers with intrahepatic cholangiocarcinomas, but it was limited by a small sample size (n = 9) of pancreatic cancer cases, according to the authors [11]. Moreover, the authors did not directly compare pancreatic cancers with cholangiocarcinomas, only each type of cancer with adjacent normal tissue [11]. The Cancer Genome Atlas project performed integrative genomic analyses including small RNA sequencing analyses of 36 cholangiocarcinoma samples [12]. Integrative clustering from TCGA data revealed the dominant role of cell-of-origin patterns [12]. These data from bulk primary tumors, however, cannot be directly used to determine the tissue-of-origin of liver metastases because of the confounding signals from the microenvironment. Hepatocyte-specific microRNAs, for example, may prevent bulk primary tumor analyses from capturing real microRNA signatures discriminating cholangiocarcinoma cells from gastrointestinal adenocarcinoma cells.

To fully address these issues, the current analyses incorporated data from in situ hybridization and cell line analyses, focusing on the identification of cholangiocarcinoma-specific microRNA profiles. More importantly, the availability of metastasectomy samples provided us with a unique opportunity to validate the predictive value of discriminatory microRNAs identified in the primary tumors. Using our genomics expertise [13, 14], we performed single-protocol microRNA profiling analyses using frozen primary tumors originating from the lung, pancreas, hepatobiliary tree, kidney, bowel, genital system, and stomach; the most common sites for carcinomas of unknown primary-origin according to autopsy studies [15]. We enriched with intrahepatic cholangiocarcinomas, which are relatively common in Korea [16]. As a result, we report microRNA signatures that could differentiate adenocarcinomas in the liver according to their tissues-of-origin.

## Materials and methods

### Tissue samples

Samples were collected at the time of surgery from patients at the National Cancer Center and the Soonchunhyang University Hospital in Korea, between 2001 to 2013. Specimens were kept frozen in liquid nitrogen until analysis. The training-sample set was composed of 195 frozen primary tumor samples comprised of 14 tumor types that were mostly adenocarcinomas (Table 1). Primary tumors included 23 intrahepatic cholangiocarcinomas (procured between 2001 and 2007), 29 colorectal adenocarcinomas, six gastric adenocarcinomas, 13 pancreatic ductal adenocarcinomas, ten HCCs, 26 lung adenocarcinomas, six small-cell lung cancers (SCLCs), 23 breast adenocarcinomas, 12 endometrial endometrioid adenocarcinomas, 11 ovarian serous adenocarcinomas, nine renal-cell carcinomas (RCCs), eight prostate adenocarcinomas, 11 thyroid papillary adenocarcinomas, and eight acute leukemias (Table 1). The test set was composed of two intrahepatic cholangiocarcinomas (procured in 2011) and 31 liver metastases originating from colon (n = 29) and ovaries (n = 2).

**Table 1:** Clinicopathological characteristics of tumor samples.

Training set				
Primary tumor	*No*.	Male	Median age(yr)	Subtype
Cholangiocarcinoma	23	15 (65%)	60	
Colorectal	29	19 (66%)	63	
Gastric	6	3 (50%)	70	intestinal 3, diffuse 3
Pancreatic	13	9 (69%)	59	ductal 13
HCC	10	9 (90%)	59	
Lung (adenocarcinoma)	26	12 (46%)	62	
Lung (SCLC)	6	5 (83%)	59	
Breast	23	0	44	HER2 16, TN 2, luminal 5
Endometrial	12	0	58	endometrioid 12
Ovarian	11	0	54	serous 11
Renal	9	4 (44%)	61	clear cell 9
Prostate	8	8 (100%)	67	
Thyroid	11	0	47	papillary 11
Leukemia	8	5 (63%)	36	ALL 5, AML 3
**Test set**				
Cholangiocarcinoma	2	2 (100%)	58	
Liver metastases	31	20 (65%)	61	colorectal 29, ovarian 2

HCC, hepatocellular carcinoma; SCLC, small-cell lung cancer; TN, triple-negative; ALL, acute lymphocytic leukemia; AML, acute myelogenous leukemia.

### MicroRNA microarrays

A 10 μm-thick top slide from tissue samples was stained with hematoxylin and eosin. Guided by this top slide, remaining tissue was macrodissected to trim non-tumorous stromal components. Macrodissected, the frozen tissue sample (>50% tumor content) was then mechanically crushed in liquid nitrogen, homogenized, and subjected to RNA isolation using TRI reagent (Thermo Fisher Scientific, Waltham, MA), according to the manufacturer’s instructions. Total RNA was then subjected to DNAse I treatment. After confirming ribosomal RNA bands were intact, we performed poly-A tailing on 500 ng of total RNA. FlashTag Biotin HSR Labeling Kit (Genisphere LLC, Hatfield, PA) was used to join a proprietary biotin-labeled dendrimer molecule to the 3′ ends using DNA ligase. Labeled samples were then hybridized to GeneChip miRNA 2.0 microarrays (Affymetrix, Santa Clara, CA) at 48°C for 16 h, washed, stained with a Streptavidin-PE solution, and scanned. GeneChip miRNA 2.0 microarrays are based on miRbase (version 15) and contain 15,644 mature microRNA probe sets from 131 organisms. All cell files were robust multi-array average (RMA)-normalized. After filtering out star-form microRNAs, we subjected 914 human microRNAs to further analyses for this study.

### Immunohistochemistry

All cases in the test set were subjected to the ImmPRESS peroxidase detection system (Vector Laboratories, MP-7401 and MP-7402) to detect CDX2, CK20, CK7, and CA125 proteins. The following antibodies were used in this study; mouse monoclonal anti-CK7 antibody (1:100; Thermo scientific, MA1-06316), rabbit monoclonal anti-CK20 antibody (1:100; Abcam, ab76126), mouse monoclonal anti-CDX2 antibody (1:100; BioGenex, MU392-UC), and mouse monoclonal anti-CA125 (1:50; Thermo scientific, MA5-11579). Briefly, frozen tissue sections were fixed with acetone for 10 min and immersed for 10 min in 3% hydrogen peroxide to block endogenous peroxidase activity. After washing in PBS, the sections were incubated in normal blocking serum provided in the kit. The sections were then incubated for 30 min at room temperature with the diluted primary antibodies. Negative controls were performed by omitting the primary antibody and diluent-substitution. The sections were then incubated with the appropriate secondary antibody conjugated with horseradish peroxidase for 30 min at room temperature. Subsequently, the sections were subjected to colorimetric detection with ImmPact DAB substrate (Vector Laboratories, SK-4105). The slides were counterstained with Mayer’s hematoxylin for 10s. Immunohistochemical evaluation was performed by two pathologists who were blinded to any clinical information. The nuclear staining for CDX2 and cytoplasmic staining for CK20, CK7, and CA125 were assessed in the tumor cells, and scored according to the percentage of positively stained tumor cells: negative, less than 5%; equivocal, from 5–50%; positive, more than 50%.

### Statistical analyses

**Fig 1:**
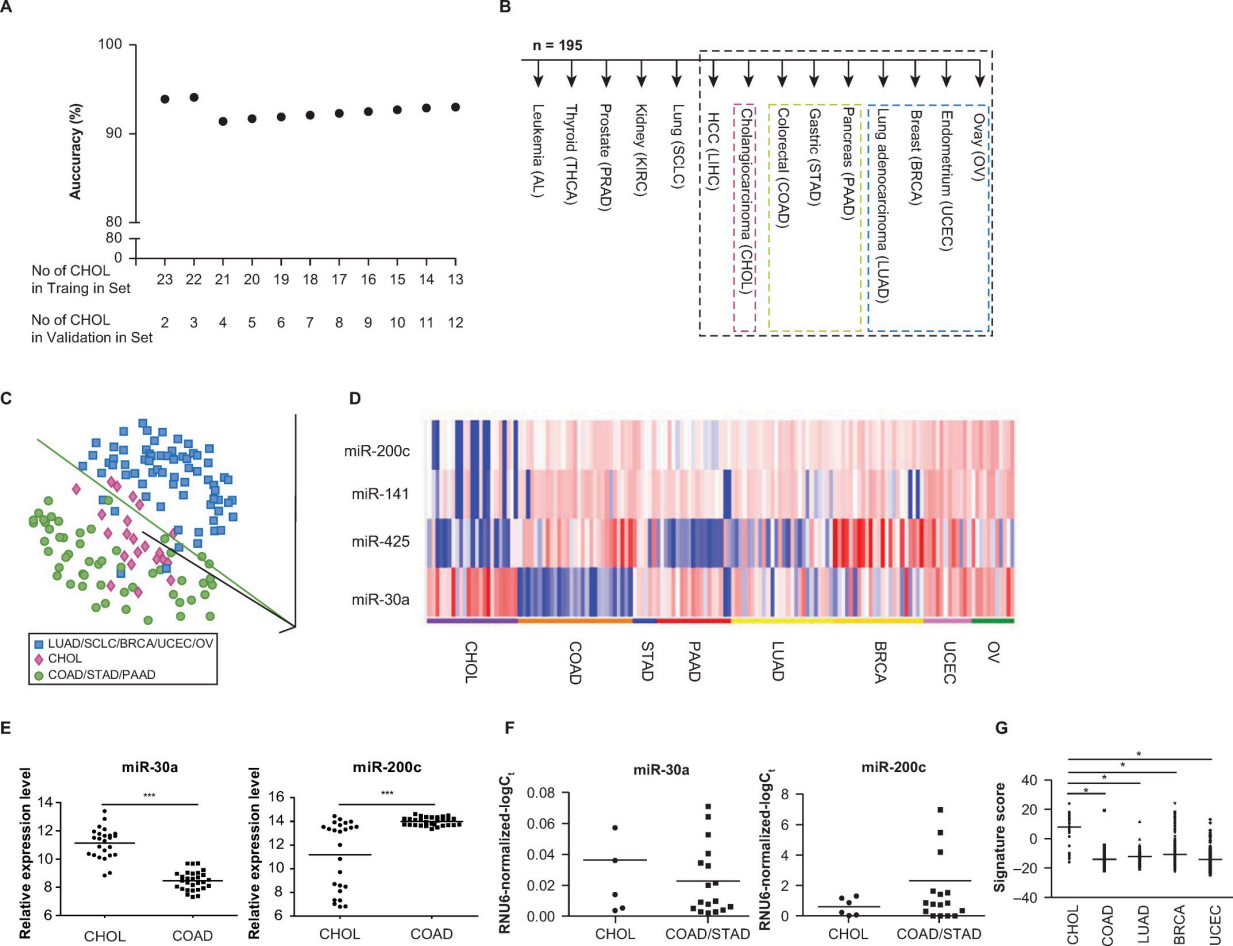
MicroRNAs for tissue origin. (A) Prediction accuracies of 1-NN analyses on the test set at feature selection p < 10^−10^, after allocating different numbers of cholangiocarcinomas to the training set according to chronological order (B) Decision tree analysis. MicroRNAs that were differentially expressed at feature selection p cutoff of 0.00005 were used to predict the tissue of origin at each node of the decision tree. Samples for node no. 7 (surrounded by black broken lines) were further evaluated in Fig 1C and 1D. (C) PCA plot for samples in node no. 7 of (B), based on 914 microRNAs. Each sphere represents each sample and ‘1-correlation’ was used as a distance metric. Cholangiocarcinomas (shown in red), gastrointestinal/pancreatic cancers (shown in green), and non-digestive system cancers (shown in blue) clustered separately. (D) Expression profiles for microRNAs in node no. 7 of (B) in the training set. Expression profiles of 3 microRNAs underexpressed in cholangiocarcinomas compared with extrahepatic cancers in digestive and non-digestive systems (upper panel) and 1 overexprexpressed microRNAs (lower panel) at feature selection p < 0.00005. Since the apparent overexpression of miR-122 in cholangiocarcinomas is presumably due to contaminating hepatocytes in the sample, we decided to exclude miR-122 from a set of discriminatory microRNAs comprising the node no. 7 of the decision tree. A heatmap generated using a log2-pseudocolor image with microRNA centering. Red and blue colors denote high and low expression of microRNAs, respectively. A scale bar for the log2-expression is shown at the bottom. (E) Expression profiles of miR-30a and miR-200c between 25 intrahepatic cholangiocarcinomas (in the training and test sets) and 29 colorectal cancer metastases in the test set. Scatter plots display median microarray signal values (***p < 10^−14^ and p < 10^−5^; *t*-test). (F) Real-time RT-PCR profiles of miR-30a and miR-200c in cell lines. Scatter plots display median values of RNU6-normalized–log_2_ Ct values (p = 0.22 and p = 0.30, respectively; *t*-test). (G) Expression profiles of cholangiocarcinoma signature in TCGA small RNA sequencing data. Scatter plots display median values of normalized RPKM (*p < 10^−10^; t-test).

RMA-summarized microarray data were analyzed using BRB-Arraytools software (NCI, Bethesda, MD) [17]. Principal component analyses (PCA) were performed using 1-correlation as a distance metric. The 1-nearest neighbor (1-NN) algorithm and differentially-expressed microRNAs was used for class prediction. We predicted the primary tumor tissue-of-origin by applying the 1-NN classifier to the test set composed of metastasectomy samples and cholangiocarcinomas. MicroRNAs that were differentially expressed in the training subset were employed to predict the tissue-of-origin in the test set. Binary decision tree analyses were also used to build a microRNA model for predicting tissue-of-origin. Branches were selected at each node of the decision tree using the 1-NN classifiers and microRNAs that were differentially expressed between two tumor types (Fig 1B).

### The Cancer Genome Atlas (TCGA) data analysis

TCGA small RNA sequencing data were downloaded from the Genomic Data Commons Data Portal (GDC, http://protal.gdc.cancer.gov) and normalized by z-scores. The cholangiocarcinoma signature score was calculated based on the weighted average of the normalized reads per kilobase of transcript, per million mapped reads (RPKM) of five microRNAs for each tumor.

### Ethics statement

National Cancer Center institutional review board waived the requirement for informed consent to participate in this study (NCCNCS12633). All data/samples were fully anonymized and the medical records were accessed from Sep 2012 to Sep 2014.

## Results

### Nearest neighbor predictions based on 195 primary tumors

When microRNAs that were differentially expressed among the 195 primary tumors in the training set were applied to the test set of liver metastasectomy samples, the prediction accuracy was consistently 93.9% at p values ranging from 10–4 to 10–10. Using 229 microRNAs that were differentially expressed among tumor types at p < 10^−10^ (S1 Table), 93.9% of the test set samples (31 of 33) were correctly identified for tissue-of-origin. There were two misclassified samples: an ovarian cancer (OV) metastasis sample (predicted to be a breast cancer metastasis) and a colorectal cancer metastasis (predicted to be a gastric cancer metastasis).

These results were obtained when 23 cholangiocarcinoma samples (procured between 2001 and 2007) were allocated to the training set, and two cholangiocarcinoma samples (procured in 2011) were allocated to the test set. To rule out a possibility of overfitting, we tested the performance of our discriminatory microRNAs using various class labeling schemes. First, we allocated different numbers of cholangiocarcinomas to the training set according to chronological order and performed the same 1-NN predictions for the test set (at feature selection p < 10^−10^). The overall prediction accuracies were 91.4% or higher for various training-to-test allocation schemes (Fig 1A). Second, we conducted the same 1-NN class prediction analyses by randomly dividing a whole set of 25 cholangiocarinoma samples into two (training/test) subsets at 2-to-1 ratio. When we evaluated the prediction accuracy of the 1-NN prediction at feature selection p < 10^−10^ for each random test set, the median prediction accuracy was 90% (range, 81.0─94.7) in 100 random datasets. Thus, high (≥ 90%) prediction accuracies throughout various class labeling schemes indicate the robustness of our tissue-specific discriminatory microRNAs in predicting the tissue-of-origin for adenocarcinomas in the liver.

### Decision tree analyses

#### Leukemia, thyroid, prostate, renal, neuroendocrine, and hepatocellular cancers (nodes no. 1–6)

To enhance the potential clinical utility of our microRNA profiles, we also employed a binary decision tree-based classification with some modification of similar schemes. In this approach, the tissue-of-origin was assigned by selecting one of the two branches at each node using the 1-NN algorithm, in order to predict the primary tissue origins of the metastases, especially the liver metastases. Branches were selected at each node of the decision tree using microRNAs that were differentially expressed between two tumor types.

**Fig 2:**
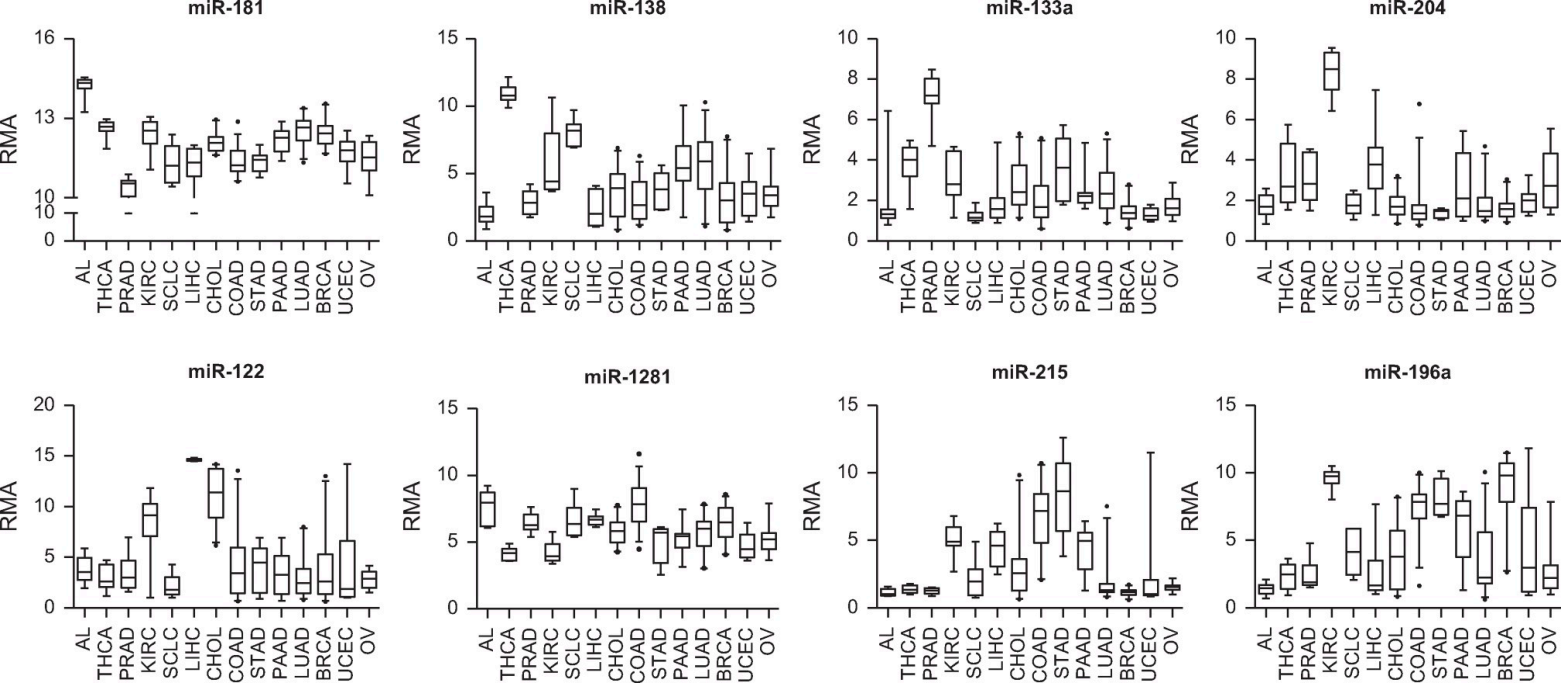
Expression profiles of selective discriminatory microRNAs of each node in the training set. Discriminatory microRNAs were defined as microRNAs differentially expressed between two branches at each node of the decision tree at p < 0.00005. The tissue of origin was assigned by selecting one of the two branches at each node using these discriminatory microRNAs. Acute leukemia (AL), thyroid cancer (THCA), prostate adenocarcinoma (PRAD), renal cell carcinoma (KIRC), small cell lung carcinoma (SCLC), hepatocellular carcinoma (LIHC), cholangiocarcinoma (CHOL), colorectal adenocarcinoma (COAD), gastric adenocarcinoma (STAD), pancreatic adenocarcinoma (PAAD), lung adenocarcinoma (LUAD), breast adenocarcinoma (BRCA), uterine endometrial carcinoma (UCEC), ovarian cancer (OV).

**Fig 3:**
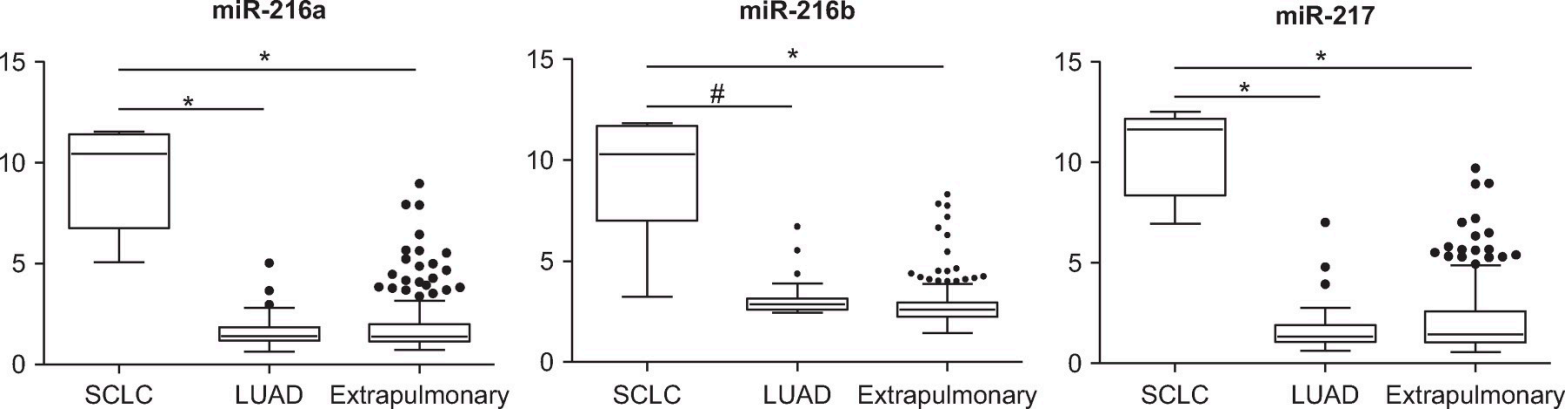
Discriminatory microRNAs for small cell lung cancer. Discriminatory microRNAs comprising the node no. 6 of the decision tree that differentiate small cell lung cancer (SCLC) from primary lung adenocarcinoma (LUAD) and extrapulmonary cancers (*p < 10^−10^; #p = 2 x 10^−9^; *t-*test).

According to our unsupervised PCA analysis, leukemias, thyroid, prostate, RCCs, SCLCs, and HCCs formed their own distinct clusters. As initial steps in the decision tree scheme, each of these five tumor types, with distinct microRNA profiles, was differentiated from the rest of the samples using differentially expressed microRNAs at feature selection of p < 0.00005 between the two groups diverging from each node (Fig 1B). The discriminatory microRNAs at each node of the decision tree are summarized in S2 Table. The miR-181 family, which was much more abundant in leukemia than in solid tumors [18], was the most characteristic microRNA at node no. 1 (leukemia *vs*. non-leukemia) of the decision tree (Fig 2). Thyroid-specific miR-138 and miR-146b-5p were most characteristic at node no. 2 (thyroid *vs*. non-thyroid) [19]. At node no. 3 (prostate *vs*. non-prostate), prostate cancer was distinguished from the other tumors by the overexpression of miR-133a and miR-133b (Fig 2) [20]. Overexpressions of miR-204 and miR-122 in RCCs and HCCs most were characteristic at nodes no. 4 (RCC *vs*. non-RCC) and no. 6 (HCC *vs*. non-HCC), respectively (Fig 2) [21, 22]. Of note, miR-216 and miR-217 were overexpressed in SCLC (Fig 3).

#### Intrahepatic cholangiocarcinoma (node no. 7)

At node no. 7 (cholangiocarcinoma *vs*. non-cholangiocarcinoma) of the decision tree, there were five differentially expressed microRNAs between cholangiocarcinomas and extrahepatic cancers of digestive and non-digestive systems at p < 0.00005. When solitary lesions are found in patients with known primary tumors, a liver biopsy is usually performed to distinguish between primary and metastatic liver cancers, given that metastasetomy is indicated only in selected clinical setting [23–25]. However, unlike HCC, intrahepatic cholangiocarcinoma is an adenocarcinoma that histologically resembles tumors originating from the pancreas, stomach, or colon, and currently lacks any validated, tissue-specific immunohistochemistry markers (Fig 1B). Given the differences in treatment strategy (local versus systemic) for these two diseases, there is an important unmet clinical need to develop a method for distinguishing between them.

At node no. 7, the overexpression of miR-122 and miR-30a, and the underexpression of the miR-200 family (miR-200c and miR-141), were most characteristic for cholangiocarcinoma. However, miR-122 is expressed at an extremely high level in both normal liver and in HCCs [22, 26], but at a relatively low level in cholangiocarcinoma cell lines [27]. We therefore reasoned that the apparent overexpression of miR-122 in cholangiocarcinomas might be primarily due to contaminating hepatocytes in bulk tumor samples, and tested for this possibility using in situ hybridization experiments. Indeed, in situ assessment of miR-122 demonstrated a very low expression of it in cholangiocarcinomas and a moderate-strong expression of it in HCCs (S1 Fig). Since the level of miR-122 expression in bulk tumor cannot differentiate between cholangiocarcinoma and liver metastasis, we decided to exclude miR-122 from a set of discriminatory microRNAs characterizing node no. 7 and defined the remaining four microRNAs as a cholangiocarcinoma signature (Table 2 and Fig 1D).

**Table 2:** The *cholangiocarcinoma signature* for the differential expression of microRNAs between intrahepatic cholangiocarcinomas and extrahepatic adenocarcinomas originating from the colon, stomach, pancreas, lung, breast, uterus, and ovary, at p < 0.00005 (node no. 7 of the decision tree; miR-122 was omitted from the list.).

***Increased in cholangiocarcinoma***					
Probe set	p	t-value	CHOL^1^	Extrahepatic	FC^2^
miR-30a	3.7 × 10^−5^	4.26	11.12	9.55	1.57
***Decreased in cholangiocarcinoma***					
Probe set	p	t-value	CHOL^1^	Extrahepatic	FC^2^
miR-200c	<1 × 10^−7^	-9.101	10.87	13.84	-2.97
miR-141	<1 × 10^−7^	-6.434	4.79	9.12	-4.33
miR-425	2 × 10^−6^	-4.937	10.5	11.31	-0.81

^**1**^CHOL, cholangiocarcinoma

^**2**^FC, expression fold change of cholangiocarcinoma to extrahepatic adenocarcinomas.

Most prominent in this cholangiocarcinoma signature was the overexpression of miR-30a in cholangiocarcinomas (Table 2). The expression level of miR-30a of cholangiocarcinoma was higher than that of extrahepatic primary adenocarcinomas from the gastrointestinal tract, the pancreas, the lung, the breast, the uterus, and the ovary (at node no. 7), although it was lower than those of RCCs and thyroid cancers. This finding is consistent with a report that miR-30a knockdown in zebrafish larvae results in defective biliary morphogenesis [28]. Primary intrahepatic carcinomas expressed stronger miR-30a and weaker miR-200c than did colorectal cancer metastases (p < 10^−14^ and p < 10^−5^, respectively; Fig 1E). According to the quantitative real-time polymerase chain reaction (qRT-PCR), cholangiocarcinoma cell lines also exhibited a trend for miR-30a overexpression and miR-200c underexpression, as compared with colorectal and gastric cancer cell lines (Fig 1F).

#### Digestive (nodes no. 8–10) and non-digestive (nodes no. 11–13) extrahepatic primary adenocarcinomas

The cholangiocarcinoma signature enabled accurate distinctions between primary and metastatic adenocarcinomas of the liver as shown above. Clinically, once the possibility of a primary liver adenocarcinoma is ruled out, determining the tissue-of-origin for metastatic adenocarcinoma is the next step for planning management. When a tissue-of-origin was assigned by selecting one branch at each node using the 1-NN algorithm and differentially expressed microRNAs (p < 0.00005), miR-1281 overexpression in colorectal cancers was the most characteristic microRNA to differentiate them from other tumors, including gastric cancers (node no. 8 (colorectal *vs*. non-colorectal)). MiR-215 was a relatively stomach-specific microRNA and characterized node no. 9 (gastric *vs*. non-gastric). While accumulating data suggest a role for miR-215 in the development and progression of gastric cancer [29], our study is the first to suggest stomach-specificity for miR-215 in the digestive system. As previously reported, miR-194 and miR-192 were relatively abundant in both colorectal and gastric cancers compared to non-gastrointestinal tumors [30]. At node no. 12 (breast *vs*. non-breast), miR-196a was abundant in breast cancers, whereas miR-449a and miR-449b were relatively abundant in endometrial and ovarian cancers [31] (Fig 2).

#### Application of the decision tree to the test set

When the present decision tree was applied to the test set, the prediction accuracy was 84.8% (28 out of 33 samples). Four colorectal cancer metastases were misclassified as cholangiocarcinomas (n = 2), a gastric carcinoma (n = 1), and a lung adenocarcinoma (n = 1). An ovarian cancer metastasis was misclassified as a cholangiocarcinoma.

#### External validation of node no. 7

Given the important clinical relevance, the cholangiocarcinoma signature (node no. 7; Table 2) was additionally validated using small RNA sequencing data from TCGA. As metastasectomy samples were not available in dataset from TCGA, TCGA primary tumors were compared for their expression of the cholangiocarcinoma signature corresponding to the node no. 7 (cholangiocarcinoma *vs*. non-cholangiocarcinoma) of our decision tree. As in the training set, cholangiocarcinoma signatures of TCGA colorectal (n = 221), lung (n = 245), breast (n = 748), and endometrial (n = 406) adenocarcinomas were weaker than that of intra- and extra-hepatic cholangiocarcinomas in the dataset from TCGA (n = 36) (Fig 1G). Thus, cholangiocarcinoma signature, which was identified in our training set, was validated in the external dataset from TCGA.

#### Immunohistochemistry

Finally, we performed in-parallel immunohistochemical staining analyses for the test set, to evaluate the potential utility of the present microRNA profiling methods, as complementary assays for conventional immunohistochemistry (Table 3). A typical colorectal immunophenotype of CDX2+/CK20+/CK7- [32] was observed in 26 of 29 samples, but three of them were CK20-, CK7+, or had equivocal CDX2 staining, which meant that these cases could not be confidently diagnosed as being of colorectal origin (S2 Fig). These three equivocal cases were correctly predicted as having a colorectal origin by our 1-NN prediction based on microRNA profiles of 195 primary tumors, again suggesting the potential usefulness of the present microRNA profiling methods. Both primary cholangiocarcinomas in the test set showed a CK20-/CK7+/CDX2-/CA125- immunophenotype, indicating a low probability of colorectal, ovarian, and pancreatic origin [32, 33]. While these two cases could have been considered cholangiocarcinomas using immunohistochemical exclusion of other tissue origins, our microRNA assessment could also have been used clarify the immunohistochemistry diagnosis by correctly assigning them to the cholangiocarcinoma category. These results indicate that microRNA profiles may also supplement immunohistochemistry assessment in determining tissue origin for liver metastases.

**Table 3:** CDX2, CK20, CK7, and CA125 immunostaining in the test set.

	Primary tumor	CDX2	CK20	CK7	CA125
No 1	CHOL	-	-	+	-
No 2	CHOL	-	-	+	-
No 3	COAD	+	+	-	-
No 4	COAD	+	+	-	-
No 5	COAD	+	+/-	-	-
No 6	COAD	+	-	-	-
No 7	COAD	+	+	-	-
No 8	COAD	+	+	-	-
No 9	COAD	+	+	-	-
No 10	COAD	+	+	-	-
No 11	COAD	+	+	-	-
No 12	COAD	+	+	-	-
No 13	COAD	+	+	-	-
No 14	COAD	+	+	-	-
No 15	COAD	+	+	-	-
No 16	COAD	+	+	-	-
No 17	COAD	+	+	-	-
No 18	COAD	+	+	-	+/-
No 19	COAD	+	+	-	-
No 20	COAD	+	+	-	-
No 21	COAD	+	-	-	-
No 22	COAD	+	+	-	-
No 23	COAD	+	+	-	-
No 24	COAD	+	+	-	-
No 25	COAD	+	+	-	-
No 26	COAD	+	+	-	-
No 27	COAD	+/-	+	+	-
No 28	COAD	+	+	-	-
No 29	COAD	+	+	-	-
No 30	COAD	+	+	-	N/T
No 31	COAD	+	+	-	+/-
No 32	OV	N/T	-	+	+
No 33	OV	-	-	+	+/-

+, positive; -, negative; +/-, equivocal; N/T, not tested; CHOL, cholangiocarcinoma; COAD, colorectal carcinoma; OV, ovarian cancer.

## Discussion

This study suggests that microRNA profiles can distinguish between intrahepatic cholangiocarcinomas and extrahepatic cancers of the digestive system, which is often difficult due to the lack of validated tissue-specific biomarkers for cholangiocarcinoma. Cytokeratin 19, for example, is useful to differentiate cholangiocarcinoma from HCC, but its positivity is similar between cholangiocarcinoma and gastrointestinal adenocarcinoma [34]. Reports in the literature have suggested that microRNA profiling may be useful in determining tumor origin, but this profiling has not been successful in accurately distinguishing cholangiocarcinomas from metastatic liver cancers. A microRNA assay developed by Solkide et al. predicted most liver metastases to be cholangiocarcinomas, and was therefore not able to differentiate between primary and metastatic adenocarcinomas in the liver. An appreciable fraction of biliary tract and pancreatic cancers were also misclassified or vaguely predicted to be pancreatobiliary cancers in another study [3]. Whereas most of previous microRNA-based tissue-origin assays were developed based on formalin-fixed, paraffin-embedded common tumors, we chose to enrich our datasets using frozen intrahepatic cholangiocarcinomas and liver metastases in our study to avoid possible artefacts. Thinking ahead for possible clinical applications, we also minimized the number of discriminatory microRNAs in the cholangiocarcinoma signature. It should be noted that our cholangiocarcinoma signature was optimized using additional in situ hybridization experiments to increase the specificity that is often lacking in bulk tumor profiling studies.

To our knowledge, this is the first study to demonstrate that the overexpression of miR-30a and the reduced expressions of miR-200c, miR-141, and miR-425 could accurately distinguish intrahepatic cholangiocarcinomas from extrahepatic adenocarcinomas, especially in gastrointestinal cancer metastases. MiR-30a is crucial in biliary development [28], and promotes the proliferation of cholangiocarcinoma cells [35]. While miR-30a plays an oncogenic role in biliary epithelium, miR-30a acts as a tumor suppressor in prostate and gastric tissue [36, 37]. Carcinogenic role of miR-30a is therefore dependent on the tissue context. The current study is the first to leverage tissue-specific roles miR-30a for the purpose of distinguishing the tissue-of-origin of liver neoplasms. During the progression of colorectal cancer, miR-200c is overexpressed [38]. To the contrary, miR-200c is downregulated in intrahepatic cholangiocarcinoma [39], consistent with the current paper. No previous studies, however, have suggested that miR-200c could be used to differentiate intrahepatic cholangiocarcinomas from liver metastases.

In addition to the potential diagnostic role of the overexpression of miR-30a and the reduced expressions of miR-200c, miR-141, and miR-425, our study reveals several novel discoveries for the tissue-dependent microRNA expression. For example, our study is the first to reveal the overexpression of miR-216/miR-217, miR-1281, and miR-215 in SCLC, colorectal, and gastric cancers, respectively. Given the potential roles in clinical tissue-of-origin diagnosis, these novel findings warrant validation studies in the future.

Although our study is still limited by the relatively small sample size of cholangiocarcinomas, our finding was validated across the analysis platform using datasets from TCGA and cell line data. We were not able to evaluate whether these microRNAs are differentially expressed in serum samples because they were not available for this study. While the diagnosis of colorectal cancer benefits from relatively sensitive and specific biomarkers indicating a CDX2 and CK7-/CK20+ phenotype, some colorectal cancers lack such typical molecular characteristics as exemplified by our samples from colorectal cancer metastases. Notably, the present microRNA profiles were proven very useful for tissue-origin diagnosis for these equivocal colorectal metastases, suggesting their supplementary diagnostic value. Using a large number of frozen tissue samples, the current study clearly demonstrates that gastrointestinal cancer can be differentiated from cholangiocarcinomas by the 4-microRNA cholangiocarcinoma signature using miR-30a, miR-200c, miR-141, and miR-425. Although the present data requires further validation using broader and larger datasets, our results provide important clues for differentiating between adenocarcinoma tissue origins in the liver, especially for the diagnosis of gastrointestinal cancer metastases.

## Supporting information

S1 Fig Representative in situ hybridization images for miR-122 expression in cholangiocarcinoma versus HCC. (*Left panels*) A cholangiocarinoma showed no expression of miR-122, while HCC showed strong expression of miR-122 (shown in blue). U6 snRNA (middle panels) and scrambled miRNA (*Right panels)* probes were used as negative and positive control (shown in blue), respectively.
(PPTX)

S2 Fig Immunohistochemistry profiles of three colorectal cancer metastases in the test set whose tissue-of-origins were correctly predicted only by microRNA profiles. CDX2, CK20, and CK7 immunostaining profiles of these colorectal cancer metastases were not typical for colorectal cancer. No. 6 and No. 21 showed negative expression of CK20, and No. 27 displayed equivocal nuclear expression of CDX2 and strong cytoplasmic expression of CK7. These tumors were correctly assigned as colorectal cancer metastases by microRNA profiling.
(PPTX)

S1 Table Expression profile of 229 discriminatory microRNAs that were differentially expressed according to the tumor type in the training set, at feature selection p < 10^−10^. (XLSX)

S2 Table Discriminatory microRNA differentially expressed between two branches of each node of the decision tree, at feature selection p < 0.00005. (XLSX)

S3 Table TCGA small RNA sequencing data were downloaded from the Genomic Data Commons Data Portal (GDC, http://protal.gdc.cancer.gov). (XLSX)

S1 File Supplementary methods. (DOCX)
