# Supplementary material for: MiR-30a and miR-200c differentiate cholangiocarcinomas from gastrointestinal cancer liver metastases
Source: PLoS One. 2021 Apr 14;16(4):e0250083. doi: 10.1371/journal.pone.0250083 (PMC8046207; doi:10.1371/journal.pone.0250083)
Supplement: S1 Fig — (Left panels) A cholangiocarinoma showed no expression of miR-122, while HCC showed strong expression of miR-122 (shown in blue). U6 snRNA (middle panels) and scrambled miRNA (Right panels) probes were used as negative and positive control (shown in blue), respectively. (PPTX) [file pone.0250083.s001.pptx]

## Slide 1
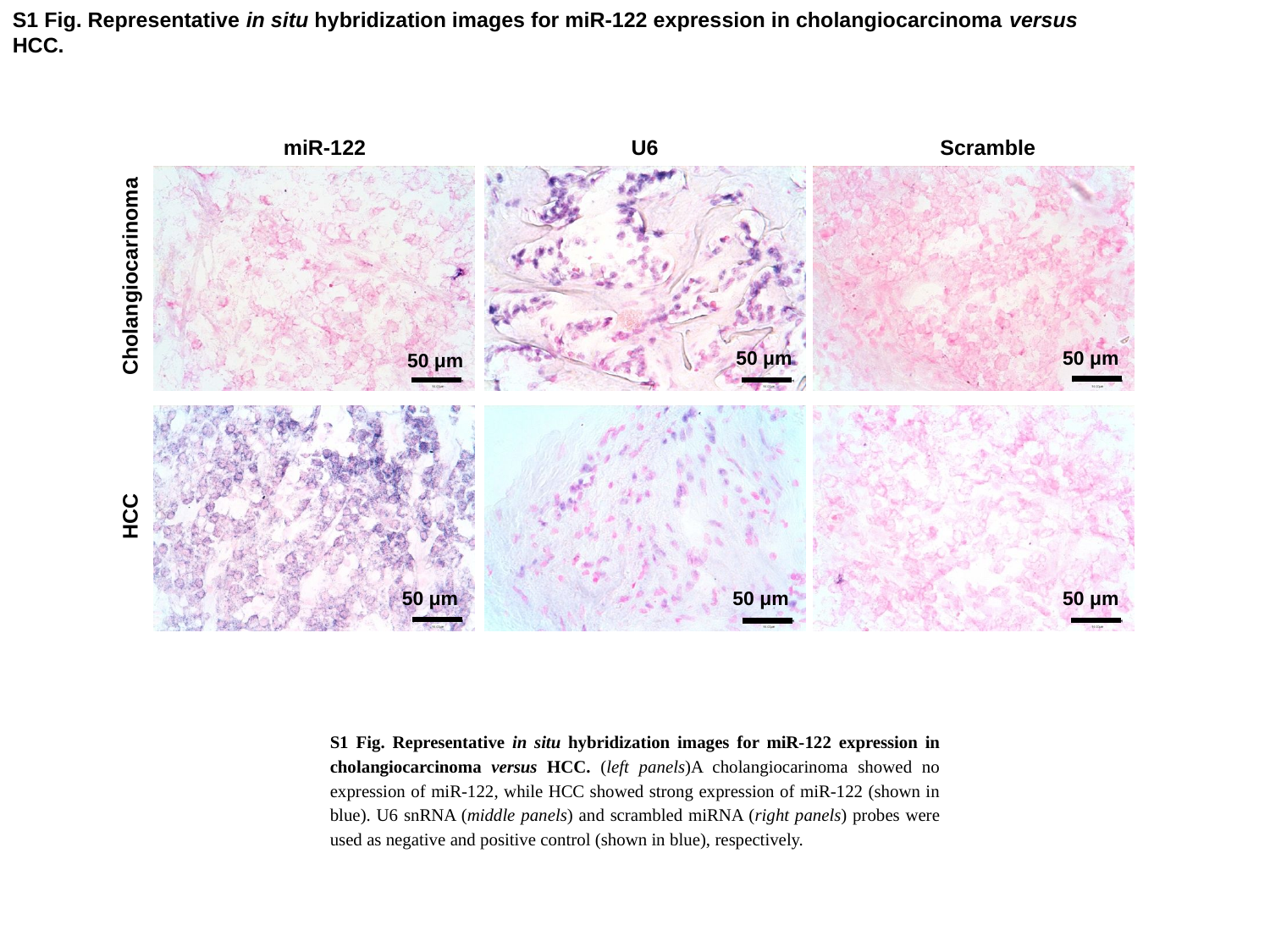

S1 Fig. Representative in situ hybridization images for miR-122 expression in cholangiocarcinoma versus HCC.
miR-122
U6
Scramble
Cholangiocarinoma
50 μm
50 μm
50 μm
HCC
50 μm
50 μm
50 μm
S1 Fig. Representative in situ hybridization images for miR-122 expression in cholangiocarcinoma versus HCC. (left panels)A cholangiocarinoma showed no expression of miR-122, while HCC showed strong expression of miR-122 (shown in blue). U6 snRNA (middle panels) and scrambled miRNA (right panels) probes were used as negative and positive control (shown in blue), respectively.
