# Supplementary material for: MiR-30a and miR-200c differentiate cholangiocarcinomas from gastrointestinal cancer liver metastases
Source: PLoS One. 2021 Apr 14;16(4):e0250083. doi: 10.1371/journal.pone.0250083 (PMC8046207; doi:10.1371/journal.pone.0250083)
Supplement: S2 Fig — CDX2, CK20, and CK7 immunostaining profiles of these colorectal cancer metastases were not typical for colorectal cancer. No. 6 and No. 21 showed negative expression of CK20, and No. 27 displayed equivocal nuclear expression of CDX2 and strong cytoplasmic expression of CK7. These tumors were correctly assigned as colorectal cancer metastases by microRNA profiling. (PPTX) [file pone.0250083.s002.pptx]

## Slide 1
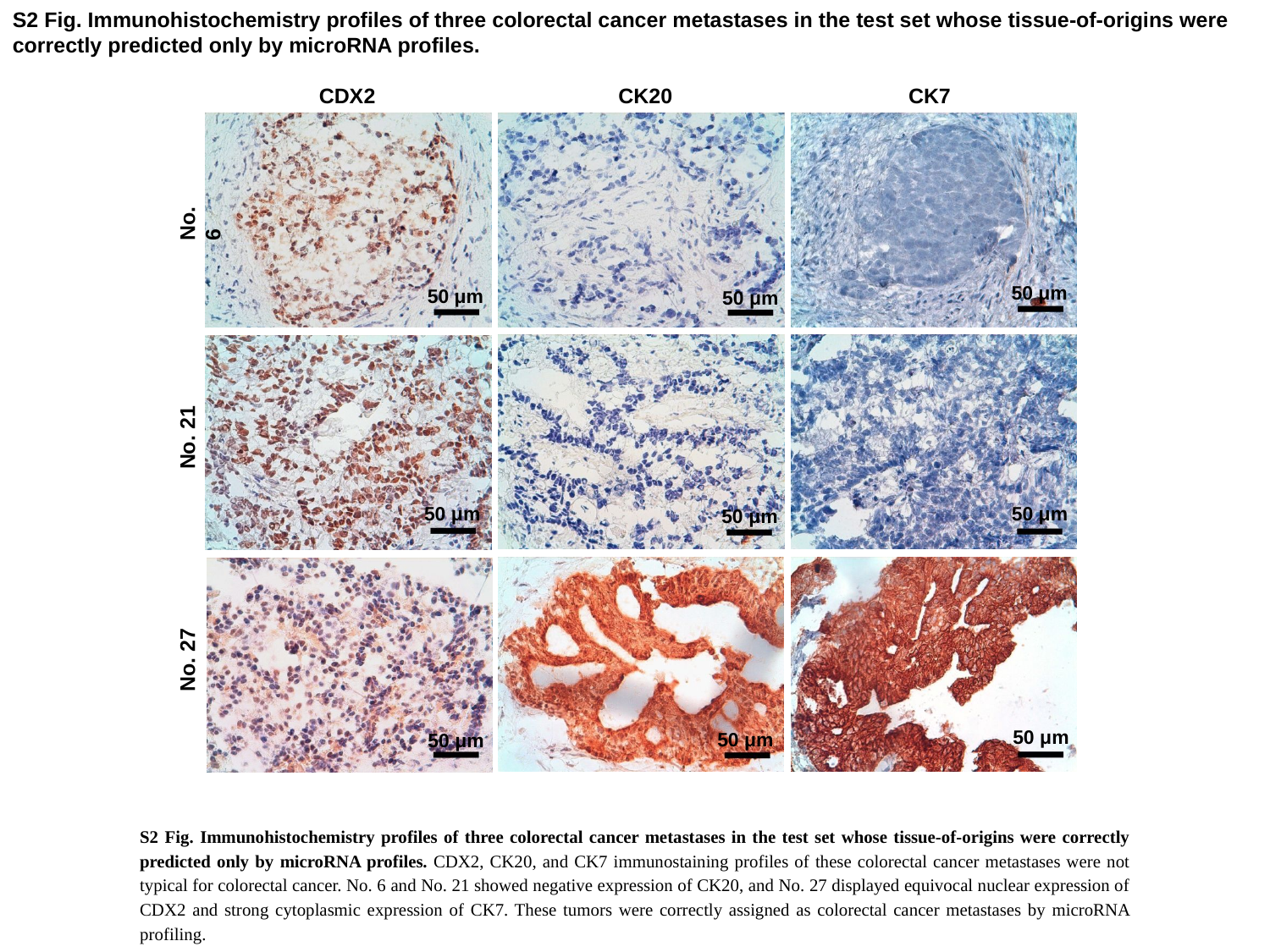

S2 Fig. Immunohistochemistry profiles of three colorectal cancer metastases in the test set whose tissue-of-origins were correctly predicted only by microRNA profiles.
CDX2
CK20
CK7
No. 6
50 μm
50 μm
50 μm
No. 21
50 μm
50 μm
50 μm
No. 27
50 μm
50 μm
50 μm
S2 Fig. Immunohistochemistry profiles of three colorectal cancer metastases in the test set whose tissue-of-origins were correctly predicted only by microRNA profiles. CDX2, CK20, and CK7 immunostaining profiles of these colorectal cancer metastases were not typical for colorectal cancer. No. 6 and No. 21 showed negative expression of CK20, and No. 27 displayed equivocal nuclear expression of CDX2 and strong cytoplasmic expression of CK7. These tumors were correctly assigned as colorectal cancer metastases by microRNA profiling.
