# Supplementary material for: MiR-30a and miR-200c differentiate cholangiocarcinomas from gastrointestinal cancer liver metastases
Source: PLoS One. 2021 Apr 14;16(4):e0250083. doi: 10.1371/journal.pone.0250083 (PMC8046207; doi:10.1371/journal.pone.0250083)
Supplement: S1 File — (DOCX) [file pone.0250083.s006.docx]

**S1 File. Supplementary methods**

***In Situ Hybridization***

Exiqon miRCURY LNA^TM^ microRNA detection kits (miR-122) were purchased from Qiagen (Hilden, Germany). Experiments were performed as recommended by the manufacturer, using five intrahepatic cholangiocarcinomas, six colorectal adenocarcinomas, five pancreatic ductal adenocarcinomas, and six hepatocellular carcinomas that were randomly selected from the training set. Briefly, 10-μm frozen sections were fixed in 10% neutralized formalin overnight and deproteinated using proteinase K (15 μg/mL). Probe complementary to miR-122 was diluted to 80 μM in hybridization solution. 80 μM scrambled-miRNA probe and 1 μM U6 snRNA probe were used as negative and positive controls, respectively. Tissue sections were incubated with diluted probes for 1 h at 55°C. After washing the slides using SSC solution (Sigma, S6639) at hybridization temperature, bound probes were detected by alkaline phosphatase conjugated anti-digoxigenin Fab fragments (1:200; Roche, 11093274910) and a subsequent color reaction using NBT/BCIP reagent (Roche, 11197471001). Nuclear fast red (Vector Laboratories, H-3403) was used as a counterstain.

***Quantitative real-time polymerase chain reaction (qRT-PCR) of RNA isolated from cell lines***

Cholangiocarcinoma cell lines (SNU-245, SNU-308, SNU-478, SNU-869, SNU-1079, and SNU-1196) were purchased from the Korean Cell Line Bank (Seoul, Korea). Colorectal and gastric cancer cell lines were maintained at National Cancer Center, and included SNU-C1, HCT-15, NCI-H716, Colo-205, SNU-407, SNU-1040, KATO-III, MKN-28, MKN-45, MKN-74, NCI-N87, SNU-1, SNU-5, SNU-16, SNU-216, SNU-484, SNU-620, and SNU-668. All these cells were grown in RPMI media supplemented with 10% fetal bovine serum at 37°C in a CO_2_ incubator.

Total RNA was isolated using a mirVana miRNA isolation kit (Thermo Fisher Scientific, Waltham, MA). We used DNAse I (Qiagen)-treated total RNA (1 µg) for reverse transcription, and a portion of the reverse transcription product and miScript PCR system (Qiagen) for real-time PCR reactions with miR-30a-3p, miR-200c-3p, and RNU-6 primers (Qiagen). Each qRT-PCR reaction was performed in duplicate using 96-well plates in a LightCycler 480 Instrument II (Roche, Basel, Switzerland). Annealing was conducted at 55°C for 30 sec. Cycle threshold (Ct) values were normalized to U6 by subtraction.

***The Cancer Genome Atlas (TCGA) data sets***

BRCA: https://portal.gdc.cancer.gov/repository?facetTab=cases&filters=%7B%22op%22%3A%22and%22%2C%22content%22%3A%5B%7B%22op%22%3A%22in%22%2C%22content%22%3A%7B%22field%22%3A%22cases.project.program.name%22%2C%22value%22%3A%5B%22TCGA%22%5D%7D%7D%2C%7B%22op%22%3A%22in%22%2C%22content%22%3A%7B%22field%22%3A%22cases.project.project_id%22%2C%22value%22%3A%5B%22TCGA-BRCA%22%5D%7D%7D%2C%7B%22op%22%3A%22in%22%2C%22content%22%3A%7B%22field%22%3A%22files.access%22%2C%22value%22%3A%5B%22open%22%5D%7D%7D%2C%7B%22op%22%3A%22in%22%2C%22content%22%3A%7B%22field%22%3A%22files.data_type%22%2C%22value%22%3A%5B%22miRNA%20Expression%20Quantification%22%5D%7D%7D%5D%7D

COAD: https://portal.gdc.cancer.gov/repository?facetTab=cases&filters=%7B%22op%22%3A%22and%22%2C%22content%22%3A%5B%7B%22op%22%3A%22in%22%2C%22content%22%3A%7B%22field%22%3A%22cases.project.program.name%22%2C%22value%22%3A%5B%22TCGA%22%5D%7D%7D%2C%7B%22op%22%3A%22in%22%2C%22content%22%3A%7B%22field%22%3A%22cases.project.project_id%22%2C%22value%22%3A%5B%22TCGA-COAD%22%5D%7D%7D%2C%7B%22op%22%3A%22in%22%2C%22content%22%3A%7B%22field%22%3A%22files.access%22%2C%22value%22%3A%5B%22open%22%5D%7D%7D%2C%7B%22op%22%3A%22in%22%2C%22content%22%3A%7B%22field%22%3A%22files.data_type%22%2C%22value%22%3A%5B%22miRNA%20Expression%20Quantification%22%5D%7D%7D%5D%7D

LUAD: https://portal.gdc.cancer.gov/repository?facetTab=cases&filters=%7B%22op%22%3A%22and%22%2C%22content%22%3A%5B%7B%22op%22%3A%22in%22%2C%22content%22%3A%7B%22field%22%3A%22cases.project.program.name%22%2C%22value%22%3A%5B%22TCGA%22%5D%7D%7D%2C%7B%22op%22%3A%22in%22%2C%22content%22%3A%7B%22field%22%3A%22cases.project.project_id%22%2C%22value%22%3A%5B%22TCGA-LUAD%22%5D%7D%7D%2C%7B%22op%22%3A%22in%22%2C%22content%22%3A%7B%22field%22%3A%22files.access%22%2C%22value%22%3A%5B%22open%22%5D%7D%7D%2C%7B%22op%22%3A%22in%22%2C%22content%22%3A%7B%22field%22%3A%22files.data_type%22%2C%22value%22%3A%5B%22miRNA%20Expression%20Quantification%22%5D%7D%7D%5D%7D

LUSC: https://portal.gdc.cancer.gov/repository?facetTab=cases&filters=%7B%22op%22%3A%22and%22%2C%22content%22%3A%5B%7B%22op%22%3A%22in%22%2C%22content%22%3A%7B%22field%22%3A%22cases.project.program.name%22%2C%22value%22%3A%5B%22TCGA%22%5D%7D%7D%2C%7B%22op%22%3A%22in%22%2C%22content%22%3A%7B%22field%22%3A%22cases.project.project_id%22%2C%22value%22%3A%5B%22TCGA-LUSC%22%5D%7D%7D%2C%7B%22op%22%3A%22in%22%2C%22content%22%3A%7B%22field%22%3A%22files.access%22%2C%22value%22%3A%5B%22open%22%5D%7D%7D%2C%7B%22op%22%3A%22in%22%2C%22content%22%3A%7B%22field%22%3A%22files.data_type%22%2C%22value%22%3A%5B%22miRNA%20Expression%20Quantification%22%5D%7D%7D%5D%7D

CHOL: https://portal.gdc.cancer.gov/repository?facetTab=cases&filters=%7B%22op%22%3A%22and%22%2C%22content%22%3A%5B%7B%22op%22%3A%22in%22%2C%22content%22%3A%7B%22field%22%3A%22cases.project.program.name%22%2C%22value%22%3A%5B%22TCGA%22%5D%7D%7D%2C%7B%22op%22%3A%22in%22%2C%22content%22%3A%7B%22field%22%3A%22cases.project.project_id%22%2C%22value%22%3A%5B%22TCGA-CHOL%22%5D%7D%7D%2C%7B%22op%22%3A%22in%22%2C%22content%22%3A%7B%22field%22%3A%22files.access%22%2C%22value%22%3A%5B%22open%22%5D%7D%7D%2C%7B%22op%22%3A%22in%22%2C%22content%22%3A%7B%22field%22%3A%22files.data_type%22%2C%22value%22%3A%5B%22miRNA%20Expression%20Quantification%22%5D%7D%7D%5D%7D

UCSC:https://portal.gdc.cancer.gov/repository?facetTab=cases&filters=%7B%22op%22%3A%22and%22%2C%22content%22%3A%5B%7B%22op%22%3A%22in%22%2C%22content%22%3A%7B%22field%22%3A%22cases.project.program.name%22%2C%22value%22%3A%5B%22TCGA%22%5D%7D%7D%2C%7B%22op%22%3A%22in%22%2C%22content%22%3A%7B%22field%22%3A%22cases.project.project_id%22%2C%22value%22%3A%5B%22TCGA-UCEC%22%5D%7D%7D%2C%7B%22op%22%3A%22in%22%2C%22content%22%3A%7B%22field%22%3A%22files.access%22%2C%22value%22%3A%5B%22open%22%5D%7D%7D%2C%7B%22op%22%3A%22in%22%2C%22content%22%3A%7B%22field%22%3A%22files.data_type%22%2C%22value%22%3A%5B%22miRNA%20Expression%20Quantification%22%5D%7D%7D%5D%7D
